# Obstetrical provider knowledge and attitudes towards cell–free DNA screening: results of a cross-sectional national survey

**DOI:** 10.1186/s12884-018-1662-z

**Published:** 2018-01-23

**Authors:** Wilson V. Chan, Jo-Ann Johnson, R. Douglas Wilson, Amy Metcalfe

**Affiliations:** Department of Obstetrics and Gynecology, University of Calgary, 4th Floor North Tower - Foothills Medical Centre, 1403 29 St NW, Calgary, AB T2N 2T9 Canada

**Keywords:** NIPT, Cell-free DNA, cfDNA, Prenatal diagnosis, Screening

## Abstract

**Background:**

Cell-free DNA (cfDNA) screening has recently acquired tremendous attention, promising patients and healthcare providers a more accurate prenatal screen for aneuploidy than other current screening modalities. It is unclear how much knowledge regarding cfDNA screening obstetrical providers possess which has important implications for the quality and content of the informed consent patients receive.

**Methods:**

A survey was designed to assess obstetrical provider knowledge and attitudes towards cfDNA screening and distributed online through the Society of Obstetricians & Gynecologists of Canada (SOGC). Chi-squared tests were used to detect differences in knowledge and attitudes between groups.

**Results:**

207 respondents completed the survey, composed of 60.6% Obstetricians/Gynecologists (OB/GYN), 15.4% Maternal Fetal Medicine (MFM) specialists, 16.5% General Practitioners (GP), and 7.5% Midwives (MW). MFM demonstrated a significant trend of being most knowledgeable about cfDNA screening followed by OB/GYN, GP, and lastly MW in almost all aspects of cfDNA screening. All groups demonstrated an overall positive attitude towards cfDNA screening; however, OB/GYN and MFM demonstrated a significantly more positive attitude than GP and MW. Despite not yet being a diagnostic test, 19.4% of GP would offer termination of pregnancy immediately following a positive cfDNA screen result compared to none of the MFM and only few OB/GYN or MW.

**Conclusions:**

We have demonstrated that different types of obstetrical providers possess varying amounts of knowledge regarding cfDNA screening with MFM currently having greater knowledge to all other groups. All obstetrical providers must have adequate prenatal screening understanding so that we can embrace the benefits of this novel and promising technology while protecting the integrity of the informed consent process.

**Electronic supplementary material:**

The online version of this article (10.1186/s12884-018-1662-z) contains supplementary material, which is available to authorized users.

## Background

Non-invasive prenatal screening (NIPS) based on cfDNA in the maternal plasma has recently acquired tremendous amounts of attention, promising patients and healthcare providers a prenatal genetic screening test for aneuploidy that is more accurate than the current ultrasound and placental analyte screening modalities, as well as safer than invasive diagnostic testing. Prenatal genetic screening methods have used non-invasive maternal serum screening testing protocols such as the first trimester combined or integrated approaches, which identifies up to 95% of trisomies, with a false positive rate of 5–23% in the general population [1–3]. Given a screen positive risk result (as determined by test’s ‘cut-off’ number), the patients are then offered the option of invasive diagnostic procedures such as chorionic villus sampling or amniocentesis, which carry a 0.5–1% risk of miscarriage [4].

cfDNA screening is an important clinical advancement over alternative screening modalities as it has an overall detection rate (DR) of 99.7% and false-positive rate (FPR) of 0.04% for trisomy 21 [5]. Different professional societies, such as the Society of Obstetricians and Gynecologists of Canada (SOGC) and the American College of Obstetricians and Gynecologists (ACOG), agree that cfDNA screening is a highly effective form of early prenatal screening of common trisomies (21, 18, 13) after 10 weeks’ gestation [6, 7]. However, there are several limitations to this screening test. cfDNA screening is a rapidly evolving field and at the time when we constructed our survey, DRs for individual aneuploidies were highly discrepant. For example Trisomy 13 had a DR of 80% and FPR of 0.05%, which was significantly lower than the performance for Trisomy 21 [8]*.* A recent meta-analysis by Nicolaides et al. however, has since shown that DR and FPR for Trisomy 13 are indeed much better at 99.0% and 0.04%*,* respectively [5]. Furthermore, despite its excellent performance, cfDNA screening is not as yet considered diagnostic and it often has variable test failure rates due to insufficient cfDNA in maternal plasma, ranging anywhere from 1% to 4.6% of screened women [9, 10]. Indeed, a study looking at the opinions of healthcare professionals about cfDNA screening implementation in the UK has identified that explaining the limitations of this test and making it clear to women that positive results need to be confirmed by invasive testing, is essential to ensure informed consent [11].

Currently in Canada, offering cfDNA screening to all women as primary screening has not yet been deemed to be fiscally feasible in most provinces [7]. Instead, a contingent model with adjustable cut-offs has been recommended by the SOGC as the most cost-effective approach that could achieve a high detection rate while retaining the benefits of conventional screening based on serum and ultrasound markers [7]. Other professional societies such as the American Congress of Obstetricians and Gynecolgists (ACOG) and American College of Medical Genetics and Genomics (ACMG), are more permissive in recommending cfDNA as an initial screening option for all women who are not significantly obese [6, 12]. Despite different recommendation as to the implementation of cfDNA screening, professional societies universally agree that ensuring obstetrical providers have an adequate level of knowledge about test performance is a crucial element to the informed consent process, thus allowing patients to make educated decisions. Canada has a publicly funded health care system where obstetrical care is provided by maternal fetal medicine specialists (MFM), obstetricians-gynecologists (OB/GYN), general practitioners (GP), and midwives (MW) depending on a number of factors including a patient’s obstetrical risk, choice, and geographical location. Currently in Canada, cfDNA screening tests are offered through several different companies, require referral from a physician, and have an out-of-pocket cost to the patient of approximately $450, with the current exception women who live in the provinces of Ontario or British Columbia where provincial health insurance provides full reimbursement for the cost of cfDNA tests to high-risk women who fulfill specific screening program criteria [13, 14]. In Canada, there have been no studies looking at the knowledge and attitudes of obstetrical providers regarding cfDNA screening. Considering cfDNA screening is a novel technology with substantial implications, this lack of data about Canadian obstetrical providers raises potential questions regarding the quality and level of the informed consent patients are receiving.

In this study, we used a cross-sectional online survey to identify the attitudes towards, and knowledge of cfDNA screening between and amongst obstetrical providers in Canada, including MFM specialists, OB/GYN, GP, and MW.

## Methods

A brief online survey [see Additional file 1] was designed for health professionals to assess obstetrical provider knowledge of, and attitudes towards cfDNA screening. The survey was developed by our research team and it was composed of 3 main sections including a knowledge assessment section, an attitudes scale, and demographic questions. The knowledge portion was created by our research team who possess extensive knowledge on cfDNA screening and tackled aspects around cfDNA screening including knowledge about the conditions it is commercially available to detect, detection rates, accessibility, and other general aspects which we collectively deemed important for obstetrical providers to know when offering such screening tests. ‘Correct’ answers to knowledge-based questions were derived by reviewing the scientific literature.

The survey was pilot tested with 4 care providers (physicians, nurses, and midwives) to ensure participant understanding. Following validation, which involved implementing changes to the survey according to participant feedback during pilot testing, the survey was translated into French to permit national distribution.

A link to the English and French versions of the online survey was emailed to all clinical members of the SOGC who had consented to participating in research (*n* = 1305). A reminder email was sent two weeks later. Participants received a $5 Starbucks e-card to thank them for their participation. Upon completion of the survey, participants received a brief answer sheet for their own reference, which were based on the latest research available in the literature at the time in which we constructed the survey.

Descriptive statistics were used to characterize the responder sample demographics. Attitude scores for individual questions were inverted and summed to create a total score. Scores above or equal to 22 were classified as a positive attitude towards the use of cfDNA testing as a screening modality for patients [15]. A summary knowledge score was not generated as we aimed to assess specifically where gaps in knowledge existed and if this differed by obstetrical provider group. Chi-square tests were used to examine differences in knowledge about and attitudes towards cfDNA screening by obstetrical provider group and differences in knowledge between individuals with positive and negative attitudes towards cfDNA screening. All analyses were conducted using Stata SE version 14 (College Station, Texas). Ethics approval for this study was granted by the Conjoint Health Research Ethics Board at the University of Calgary.

## Results

The survey was distributed to 1305 individuals, of which, 207 participated, yielding a response rate of 15.9%. Of the total number of respondents, 5 individuals did not indicate their type of practice and were excluded. The demographic characteristics of the remaining 202 respondents are summarized in Table 1. MFM, OB/GYN, GP and MW represented 93% of the total number of respondents; the remaining 7% were comprised of Genetic Counsellors, Nurses, and “Other” professionals. Over 84% of respondents practiced at the staff level and approximately one-third had a patient population that was at least 75% obstetrical. The years in practice ranged from the trainee level with no years in practice to > 20 years with 46.8% of respondents having > 15 years of practice. All Canadian provinces were represented in the survey with most responses coming from Ontario (43.6%) and Quebec (20.8%).

**Table 1:** Demographic characteristics of respondents

*Characteristic*	*N (%)*
Gender	
Male	44 (21.9)
Female	157 (78.1)
Type of Practice	
Obstetrician/Gynecologist	114 (56.4)
Maternal-Fetal-Medicine	29 (14.4)
General Practitioner	31 (15.4)
Midwife	14 (7.0)
Other	14 (7.0)
Level of Practice	
Staff	170 (84.2)
Fellow	7 (3.5)
Resident	17 (8.4)
Other	8 (4.0)
Percentage of Obstetrical Patients	
100%	48 (23.9)
75–99%	12 (6.0)
50–74%	63 (31.3)
25–49%	37 (18.4)
< 25%	34 (16.9)
None	7 (3.5)
Years in Practice	
≥ 20 yrs	65 (32.0)
15–19 yrs	30 (14.8)
10–14 yrs	30 (14.8)
5–9 yrs	34 (16.8)
0–4 yrs	20 (9.9)
Trainee	24 (11.8)
Geographic Distribution	
Western Canada (BC, AB, SK, MB)	59 (29.2)
Ontario	88 (43.6)
Quebec	42 (20.8)
Maritimes (NB, NS, PEI, NL)	12 (5.9)

The further analyses focuses on those 188 professionals that practiced clinical obstetrics and who would in theory be offering cfDNA screening to their patients.

Knowledge about which conditions cfDNA analysis is commercially available to screen for varied by obstetrical provider group and is summarized in Table 2. OB/GYN and MFM were more likely to know that, in addition to Trisomy 21, cfDNA analysis screens for Trisomy 18 (*p* = 0.04) and Trisomy 13 (*p* = 0.002). Furthermore, MFMs were the most likely to also know that cfDNA analysis can screen for sex chromosome aneuploidy such as Turner Syndrome (*p* = 0.001) and microdeletion syndromes such as DiGeorge Syndrome (*p* = 0.007). As indicated by Tables 2, 21.4% of MW, 16.1% of GP, 4.4% of OB/GYN and 0% of MFM (*p* = 0.008) reported not knowing what conditions cfDNA analysis screened for.

**Table 2:** Knowledge of which conditions cfDNA screening is commercially available to detect stratified by provider type (*n* = 188)

	OB/GYN (*n* = 114) % [95% CI]	MFM (*n* = 29) % [95% CI]	GP (*n* = 31) % [95%CI]	MW (*n* = 14) % [95% CI]	*p*-value
*Correctly identified that cfDNA was able to screen for:*					
Trisomy 21 (Down Syndrome)	95.6% [89.8–98.1]	100.0%	90.3% [73.4–96.9]	85.7% [55.7–96.6]	*p* = 0.156
Trisomy 18 (Edwards Syndrome)	93.0% [86.5–96.5]	100.0%	80.6% [62.6–91.2]	85.7% [55.7–96.6]	*p* = 0.040
Trisomy 13 (Patau Syndrome)	93.0% [86.5–96.5]	96.6% [78.4–99.6]	74.2% [55.8–86.8]	71.4% [42.7–89.4]	p = 0.002
Monosomy X (Turner Syndrome)	63.2% [53.8–71.2]	86.2% [67.9–94.9]	38.7% [23.2–57.0]	42.9% [19.9–69.4]	p = 0.001
22q11.22 deletion (Di George Syndrome)	23.7% [16.7–32.4]	51.7% [33.7–69.3]	29.0% [15.6–47.5]	7.1% [0.9–39.2]	p = 0.007
*Correctly identified that cfDNA was NOT able or commercially available to screen for:*					
Spina Bifida	97.4% [92.1–99.2]	100.0%	87.1% [69.7–95.2]	85.7% [55.7–96.6]	*p* = 0.020
Hirschsprung Disease	100.0%	100.0%	100.0%	100.0%	*p* = 1.0
Cystic Fibrosis	95.6% [89.8–98.2]	100.0%	93.5% [77.0–98.4]	100.0%	*p* = 0.485
*Reported not knowing what conditions cfDNA screened for.*	4.9% [1.8–10.2]	0.0%	16.1% [6.7–33.8]	21.4% [6.7–50.9]	p = 0.008

*Abbreviations: *OB/GYN* Obstetrician/Gynecologist, *MFM* Maternal Fetal Medicine Specialist, *GP* General Practitioner, *MW* Midwife

Table 3 summarizes the knowledge regarding detection rates for cfDNA screening and how it compares to other screening and diagnostic modalities by obstetrical provider group. In a similar overall trend to Table 1, MFMs demonstrated significantly more knowledge about detection rates (DR) and capabilities of cfDNA screening, followed by OB/GYN, GP, and lastly MW. As outlined in Table 3, MFM were more likely to know that DR for different trisomies using cfDNA are not equal, that DR for Trisomy 21 via cfDNA are superior to currently available prenatal screening methods, and that the range of karyotypic abnormalities presently detected via cfDNA is much less than those findings that can be detected with conventional karyotyping techniques and microarray analysis enabled by invasive techniques such as amniocentesis.

**Table 3:** Detection rates and capabilities of cfDNA screening (n = 188)

Question:	OB/GYN (n = 114) % [95% CI]	MFM (n = 29) % [95% CI]	GP (n = 31) % [95%CI]	MW (n = 14) % [95% CI]	*p*-value
*Correctly identified that detection rates are NOT equal for different trisomies such as 13, 18, and 21 using cfDNA.*	53.5% [44.2–62.5]	82.8% [64.1–92.8]	41.9% [25.8–60.0]	14.3% [3.4–44.3]	*p* = 0.000
*Correctly identified that cfDNA has a better detection rate for Trisomy 21 than currently available prenatal screening methods such as the First Trimester Combined Test or Integrated Prenatal Screening.*	88.5% [81.1–93.2]	100.0%	67.7% [49.3–81.9]	50.0% [25.1–74.9]	p = 0.000
*Correctly identified that NOT all the chromosomal abnormalities diagnosed* via *amniocentesis can also be readily detected* via *cfDNA.*	85.0% [77.0–90.5]	96.6% [78.4–99.5]	61.3% [43.0–76.8]	71.4% [42.7–89.4]	*p* = 0.017

*Abbreviations: *OB/GYN* Obstetrician/Gynecologist, *MFM* Maternal Fetal Medicine Specialist, *GP* General Practitioner, *MW* Midwife

Table 4 identifies the care pathways that obstetrical provider groups would offer patients following a positive cfDNA screen result for trisomy 13, 18, or 21. All obstetrical provider groups were similar in recognizing that immediate treatment for the fetus should not be offered based on positive cfDNA screening results; however, there was a significantly higher proportion of GP’s (19.4%) who would offer termination of the pregnancy immediately following a positive cfDNA screen result compared to 0.0% MFM, 4.4% OB/GYN, and 7.1% of MW (*p* = 0.009). MFM (96.6%) and OB/GYN (91.2%) were also more likely to offer invasive testing following a positive cfDNA screen result compared to only 77.4% of GP and 78.6% of MW (*p* = 0.047). GP (16.1%) were most likely to report not knowing who to offer cfDNA screening to, compared to only 3.4% of MFM, 0.9% of OB/GYN, and 0.0% of MW.

**Table 4:** Patient management and counselling following a positive cfDNA screen result (n = 188)

Question:	Ob/Gyn (n = 114) % [95% CI]	MFM (n = 29) % [95% CI]	GP (n = 31) % [95%CI]	MW (n = 14) % [95% CI]	*p*-value
*When cfDNA shows a high-risk result for trisomy 13, 18, or 21….*					
- correctly identified that patients should be offered:					
Invasive diagnostic testing	91.2% [84.4–95.2]	96.6% [78.4–99.6]	77.4% [59.2–89.0]	78.6% [49.1–93.3]	p = 0.047
Genetic counselling	68.4% [59.2–76.4]	62.1% [43.1–77.9]	64.5% [46.1–79.4]	78.6% [49.1–93.3]	*p* = 0.719
- correctly identified that patients should NOT be offered:					
Immediate treatment for the baby	99.1% [93.9–99.9]	100.0%	100.0%	100.0% [3.4–44.3]	*p* = 0.884
A termination of pregnancy	95.6% [89.8–98.2]	100.0%	80.6% [62.6–91.2]	92.3% [60.8–99.1]	p = 0.009
Stem cell therapy	100.0%	100.0%	100.0%	100.0%	p = 1.0
- stated insufficient/no evidence	0.0%	0.0%	0.0%	0.0%	p = 1.0
- reported not knowing	1.8% [0.4–6.8]	0.0%	3.2% [0.4–20.4]	0.0%	*p* = 0.743
*Correctly identified that the SOGC currently recommends that cfDNA should be offered in lieu of invasive testing for women at increased risk of trisomy.*	65.0% [55.6–73.1]	86.2% [67.9–94.9]	61.3% [43.0–76.8]	71.4% [42.7–89.4]	*p* = 0.172
*Correctly identified that cfDNA should be offered to:*					
Women who had another positive prenatal screening test for trisomy.	64.0% [54.7–72.4]	72.4% [53.3–85.8]	48.4% [31.3–65.8]	50.0% [25.1–74.9]	*p* = 0.186
Women with a previous pregnancy with fetal aneuploidy.	57.9% [48.6–66.7]	69.0% [49.8–83.3]	38.7% [23.2–57.0]	57.1% [30.6–80.1]	*p* = 0.116
*Correctly identified that cfDNA should NOT be offered to:*					
All pregnant women	54.4% [45.1–63.4]	55.2% [36.8–72.3]	58.1% [40.0–74.2]	50.0% [25.1–74.9]	*p* = 0.965
Reflexively to women over 35 yrs	64.9% [55.6–73.1]	65.5% [46.4–80.6]	51.6% [34.2–68.7]	50.0% [25.1–74.9]	*p* = 0.420
Only to women who can afford it	77.2% [68.5–84.0]	82.8% [64.1–92.8]	74.2% [55.8–86.8]	92.9% [60.8–99.1]	*p* = 0.477
Only to women who would consider termination of pregnancy	75.4% [66.6–82.5]	96.6% [78.4–99.5]	71.0% [52.5–84.4]	78.6% [49.1–93.3]	*p* = 0.066
*Reported not knowing who cfDNA should be offered to.*	0.9% [0.1–6.1]	3.4% [0.5–21.6]	16.1% [6.7–33.9]	0.0%	p = 0.001

*Abbreviations: *OB/GYN* Obstetrician/Gynecologist, *MFM* Maternal Fetal Medicine Specialist, *GP* General Practitioner, *MW* Midwife

Table 5 summarizes obstetrical provider knowledge on a varied range of attributes pertaining to cfDNA screening, from accessibility to technical aspects of cfDNA assay. All groups were equally knowledgeable about cfDNA sceening not being widely accessible nor free of cost in Canada. Again, MFM demonstrated a significant trend of being most knowledgeable about the factors that are associated with low fetal fraction and thus cfDNA screening test failure, followed by OB/GYN, GP, and lastly MW. Of MFM, 100% correctly selected the earliest gestation age at which cfDNA screening can be offered, compared to 63.7% of OB/GYN, 54.8% of GP, and 50.0% of MW (*p* = 0.04).

**Table 5:** Accessibility, fetal fraction, and earliest gestational age at which cfDNA screening can be offered (n = 188)

Question:	OB/GYN (n = 114) % [95% CI]	MFM (n = 29) % [95% CI]	GP (n = 31) % [95%CI]	MW (n = 14) % [95% CI]	*p*-value
*Correctly identified that cfDNA is not widely accessible and free of cost to every woman in Canada.*	89.5% [82.3–94.0]	89.7% [71.8–96.7]	77.4% [59.1–89.0]	85.7% [55.7–96.6]	*p* = 0.405
*Correctly identified that 10 weeks is the earliest gestational age at which cfDNA can be offered.*	63.7% [54.4–72.1]	100.0%	54.8% [37.1–71.5]	50.0% [25.1–74.9]	*p* = 0.036
*Correctly identified the following factors as associated with low fetal fraction:*					
Maternal weight	44.7% [35.8–54.0]	75.9% [56.8–88.3]	38.7% [23.2–57.0]	21.4% [6.7–50.9]	p = 0.002
Crown-Rump Length	14.0% [8.7–21.8]	51.7% [33.7–69.3]	9.7% [3.1–26.6]	0.0%	p = 0.000
Gestational age	64.9% [55.6–73.2]	96.6% [78.4–99.5]	41.9% [25.8–60.0]	28.6% [10.6–57.3]	p = 0.000
*Correctly identified the following factors as NOT associated with low fetal fraction:*					
Fetal sex	97.4% [92.1–99.2]	93.1% [75.6–98.3]	96.8% [79.6–99.6]	100.0%	*p* = 0.601
Parity	99.1% [93.9–99.9]	100.0%	100.0%	92.9% [60.8–99.1]	*p* = 0.133
Reported not knowing which factors are associated with low fetal fraction.	27.2% [19.8–36.2]	0.0%	48.4% [31.3–65.8]	64.3% [36.5–84.9]	p = 0.000

*Abbreviations: *OB/GYN* Obstetrician/Gynecologist, *MFM* Maternal Fetal Medicine Specialist, *GP* General Practitioner, *MW* Midwife

Our survey demonstrated that the majority of obstetrical providers (76.3%, 70.0–81.7) showed support for the use of cfDNA screening. However, a significant difference was observed between obstetrical provider groups with 82.8% (64.1–92.8) of MFM, 82.5% (74.3–88.4) of OB/GYN, 58.1% (40.0–74.2) of GP and 42.9% (19.9–69.4) of MW showing support for the use of cfDNA screening (*p* = 0.01). We found a direct association between support for cfDNA screening and knowledge of cfDNA screening. Individuals who showed support towards cfDNA screening were also more likely to correctly identify which conditions cfDNA screening does and does not screen for (*p* = 0.004 to *p* < 0.001), to know that cfDNA screening has a better detection rate for trisomy 21 than other prenatal screening tests (p < 0.001), that invasive testing should be used to confirm the results of a positive cfDNA screen result (p = 0.01), that not all chromosomal abnormalities that can be detected via amniocentesis can be screened for using cfDNA screening (*p* = 0.005), and that the level of the fetal fraction of the maternal plasma cell free DNA varies with gestational age (*p* < 0.001).

## Discussion

Given the rapid development and implementation of cfDNA screening into clinical use, it is unclear how much knowledge obstetrical providers in Canada possess about the performance and limitations of this screening test. This concern raises potential questions regarding the quality of the informed consent that patients undergoing cfDNA screening receive, since test results can have meaningful implications for the current pregnancy. Indeed, the training and ongoing education of healthcare providers about cfDNA screening has been identified as an urgent and important priority [11, 16]. Nonetheless, these screening tests have been commercially available and aggressively marketed to patients since 2011 in the US and in Canada since 2013 [16, 17]. Studies of healthcare provider attitudes done months prior to commercial availability of cfDNA screening in the U.S. showed that 85% of respondents, comprised of mostly MDs, reported not having a high level of knowledge about cfDNA screening and that 70% would follow the guidance of professional societies like ACOG [18]. Similar studies assessing Obstetricians/Gynecologists completed 1 year after commercial availability in the U.S. found that 32% of respondents had already incorporated cfDNA screening into their practice, and an additional 22% were familiar with published clinical data, while 8% had never heard of this type of technology [19]. A study looking at the attitudes and knowledge of MFM Fellows in the U.S. regarding cfDNA screening found that > 75% are comfortable ordering the test [20].

In this study we identified significant differences in attitudes towards and knowledge of cfDNA screening between obstetrical provider groups in Canada. MFMs were significantly more knowledgeable than all other provider groups in virtually all of the aspects regarding cfDNA screening, including conditions it is commercially available to screen for, detection rates nuances, and test failure rates. OB/GYN compared relatively well after MFM, followed by GP and MW in their knowledge of cfDNA screening. This is in keeping with previous findings where even within a group of only USA-based MFM Fellows, the accuracy of a MFM fellow’s knowledge regarding possible indications for cfDNA screening trended towards higher levels with increasing year in the MFM fellowship [20]. As a whole, the majority of our Canadian participants demonstrated support towards use of cfDNA screening, a similar finding to attitudes of healthcare providers found in other countries like the USA, United Kingdom, Netherlands, and China [11, 20–22]. However, we discovered that MFM and OB/GYN demonstrated significantly more support towards the use of cfDNA screening than GP and MW. We noted to be a direct association between demonstrating support for the use of cfDNA screening and knowledge of cfDNA screening. This finding is similar to a recent study assessing Dutch MW, a system where MW provide aneuploidy counselling for up to 85% of pregnant women [21]. Indeed, healthcare provider’s attitudes have been shown to affect how test informed consent is presented to patients and how it may influence patient choice/test uptake [23].

Emerging data is suggesting that test failure might be associated with an increased risk of chromosomal abnormalities [24]. This is an important piece of knowledge an obstetrical provider should share with any patient who has a ‘no result’ test and encourage repeat screening or proceeding with diagnostic testing. While many professional organizations including ACOG, SOGC, and ISPD recommends to obstetrical providers that no irretrievable obstetrical decision should be made with a positive cfDNA screen result without invasive diagnostic testing, 19.4% of GP would offer termination of pregnancy immediately following a positive cfDNA screen result compared to none of the MFM and only few OB/GYN or MW. Interestingly, in a recent survey of US obstetricians regarding cfDNA screening use, nearly 15% also misunderstood cfDNA screening as being a diagnostic test for fetal aneuploidy [25]. In a recent study looking at patient choices following a positive screen for aneuploidy by cfDNA, researchers noted that 19.6% of the patients that elected to terminate a singleton pregnancy or selectively reduce a twin pregnancy due to a suspected autosomal trisomy, did so without karyotypic confirmation [26]. Despite counselling by certified genetic counsellors in 95% of these patients where emphasis on professional recommendations to have cfDNA results confirmed before any irretrievable obstetrical decision is to be made, elective termination was still pursed [26]. It is important to note that the overall rate of pregnancy termination from cfDNA as a screening method was not found to be increased compared to previous testing methods [26]. Confirming positive cfDNA screen results by invasive diagnostic procedures is a professional recommendation, however, obstetrical providers should ultimately respect the individual choices made by patients whether to terminate or continue a pregnancy irrelevant of the presence or absence of confirmatory tests, as long as informed consent is attained.

The body of knowledge assessing the knowledge and attitudes of obstetrical providers towards cfDNA screening has expanded significantly in recent years. These studies have identified deficiencies in the current model that we use to counsel patients providing possible targets for future improvement in the knowledge translation process. For example, in a recent study looking at the US prenatal providers’ reflections on cfDNA screening, providers felt they did not have enough time to adequately counsel and educate patients and that they needed additional education about cfDNA screening which would preferably come from sources like professional societies, labs, and publications [27]. Palomaki et al. recently assessed the experience of the obstetrical care providers, as well as the 2691 women that cfDNA screening was offered to [28]. In this study, despite providers feeling positive about the ease of offering cfDNA screening and > 95% of patient feeling they had sufficient time to talk to their providers and have their questions answered, 15% mistakenly thought the test identified all genetic problems and 13% thought the test definitively ruled out Down Syndrome [28]. In combination with this growing body of knowledge, our findings could be very useful in developing educational tools for the continuing education of obstetrical care providers around the subject of cfDNA screening thus improving the quality of the informed consent process.

Our study has certain limitations. We achieved a response rate of only 15.9%, a smaller response to similar studies performed where physician response rates were approximately 35–42% [11, 18, 20, 29]. However, despite the lower response rate, we achieved higher absolute numbers of respondents (*n* = 207) than many other studies previously performed in the subject. While this study clearly identifies obstetrical provider knowledge gaps, it does not inquire further into where and how respondents gathered their current knowledge of cfDNA screening and identify possible points of intervention for future educational tools. In contrast, Swaney et al. defined formal educational activities, self-review of the literature, and discussion with peers to be the most used methods for MFM Fellows to inform themselves on cfDNA screening. Furthermore, after distribution of the survey we noted post hoc that there was an erratum in our survey in a question exploring provider knowledge about the factors that can be associated with low fetal fraction in maternal blood. We had incorrectly listed maternal cigarette smoking as being associated with low fetal fraction when in fact, the median fetal fraction has been shown to be increased by 7.5% in smokers and other groups have more recently shown maternal smoking not to affect the amount of cfDNA in maternal plasma [30, 31]. Nonetheless, this study is a reasonable representation of the knowledge and attitudes of highly experienced obstetrical provider groups across Canada. Our respondent group was reflective of the actual make-up of the SOGC from which we drew our study sample (representing MFM, OB/GYN, GP, and MW in similar proportions).

## Conclusions

This study has important implications for the obstetrical provider and the quality and content of the informed consent process when counselling patients about cfDNA screening. Indeed provider knowledge and patient autonomy are key aspects of the informed consent process in genetics screening. We have clearly demonstrated that different types of obstetrical providers possess varying amount of knowledge regarding cfDNA screening with MFM having greater knowledge to all other groups, at the present time. All maternity care providers must have adequate prenatal screening understanding, as MFM and OB/GYN are typically not the first point of contact for the majority of patients when being offered cfDNA screening. As we go forward, it is important we evaluate knowledge gaps and provide learning tools to all obstetrical providers so that we can embrace the benefits of this novel and promising technology while protecting the integrity of the informed consent process.

## Additional files

Additional file 1: Obstetrical Care Provider Survey. This file contains the sample survey which we sent to obstetrical care providers exploring their knowledge and attitudes towards cell-free DNA screening. This document includes both English and French versions (DOCX 119 kb)

## Abbreviations

ACMG: American College of Medical Genetics and Genomics
ACOG: American Congress of Obstetricians and Gynecolgists
cfDNA: Cell-free DNA
DR: Detection rate
FPR: False-positive rate
GP: General Practitioner
MFM: Maternal Fetal Medicine
MW: Midwives
NIPS: Non-invasive prenatal screening
OB/GYN: Obstetricians/Gynecologists
SOGC: Society of Obstetricians and Gynecologists of Canada
