# Supplementary material for: Obstetrical provider knowledge and attitudes towards cell–free DNA screening: results of a cross-sectional national survey
Source: BMC Pregnancy Childbirth. 2018 Jan 23;18:40. doi: 10.1186/s12884-018-1662-z (PMC5781306; doi:10.1186/s12884-018-1662-z)
Supplement: Supplementary file 1 — Obstetrical Care Provider Survey. This file contains the sample survey which we sent to obstetrical care providers exploring their knowledge and attitudes towards cell-free DNA screening. This document includes both English and French versions (DOCX 119 kb) [file 12884_2018_1662_MOESM1_ESM.docx]

**Additional File 1** – Obstetrical Care Provider Survey (English/*French* Version)

Thank you for taking part in our survey. This survey will explore your knowledge and attitudes as an antenatal care provider in Canada towards a relatively new mode of Non-Invasive Prenatal Testing (NIPT) for fetal aneuploidy, which uses cell-free DNA (cfDNA) found in maternal serum. Your participation will only require approximately 5 minutes and will remain anonymous. If you chose to provide an email address at the end of the survey, you will be sent a $5 Starbucks e-gift card. Your email address will not be tied to your survey responses.

*Merci de participer à notre sondage. Celui-ci vise à examiner vos connaissances et votre opinion en tant que prestataire de soins prénataux au Canada, au sujet d’un nouveau mode de diagnostique prénatal non invasif (DPNI), visant à détecter l’aneuploïdie fœtale grâce à l’ADN sans cellule (cfADN) prélevé dans le sérum maternel. Votre participation prendra environ cinq minutes et si vous choisissez de nous fournir une adresse courriel à la fin de ce sondage, vous recevrez une carte cadeau de 5$ pour les cafés Starbucks. Votre adresse courriel ne sera pas associée aux réponses que vous nous aurez fournies dans ce sondage.*

1. NIPT is widely accessible and free of cost to every woman in Canada.

a. True

b. False

c. Province Dependent

d. I don’t know

1. *Au Canada, le DPNI est largement accessible et gratuit pour toutes les femmes.*
2. *Vrai*
3. *Faux*
4. *Dépend de la province*
5. *Ne sais pas*

2. Which of the following conditions is NIPT commercially available to detect? **Select all that apply.**

a. Trisomy 21 (Down Syndrome)

b. Trisomy 18 (Edwards Syndrome)

c. Trisomy 13 (Patau Syndrome)

d. Spina Bifida

e. Turner Syndrome (45X)

f. Hirschsprung Disease

g. 22q11.22 deletion syndrome (DiGeorge syndrome)

h. Cystic fibrosis

i. I don’t know

1. *La ou lesquelles des maladies suivantes le DPNI disponible sur le marché permet-il de dépister ?* ***Sélectionnez toutes les réponses qui s’appliquent.***

*a. Trisomie 21 (syndrome de Down)*

*b. Trisomie 18 (syndrome d’Edwards)*

*c. Trisomie 13 (syndrome de Patau)*

*d. Spina Bifida*

*e. Syndrome de Turner (45X)*

*f. Maladie de Hirschsprung*

*g. Micro délétion 22q11 (syndrome de DiGeorge)*

*h. Fibrose kystique*

*i. Ne sais pas*

3. Detection rates are equal for different trisomies such as 13, 18, and 21 using NIPT.

a. True

b. False

c. I don’t know

*3. Le DPNI parvient à dépister de manière égale les trisomies 13, 18, et 21.*

*a. Vrai*

*b. Faux*

*c. Ne sais pas*

4. NIPT has a better detection rate for trisomy 21 than currently available prenatal screening methods such as the First Trimester Combined Test or Integrated Prenatal Screening.

a. True

b. False

c. Insufficient evidence or no evidence

d. I don’t know

*4. Le DPNI obtient un taux de dépistage de la trisomie 21 plus élevé que les méthodes de dépistage prénatal disponibles à l’heure actuelle, comme le test du premier trimestre ou le dépistage intégré (DPI).*

*a. Vrai*

*b. Faux*

*c. Preuves insuffisantes ou manque de preuves*

*d. Ne sais pas*

5. When NIPT shows a high-risk result for trisomy 13, 18, or 21, which of the following options

should patients be offered? **Select all that apply**.

a. Immediate treatment for the baby

b. A termination of pregnancy

c. Invasive diagnostic testing

d. Stem cell therapy

e. Genetic counselling

f. Insufficient evidence or no evidence for any of the above treatment options.

g. I don’t know

*5. Lorsque le DPNI détecte un risqué élevé de trisomie 13, 18 ou 21, laquelle des options suivantes devrait-elle être proposée aux patientes ?* ***Sélectionnez toutes les réponses qui s’appliquent.***

*a. Traitement immédiat pour le bébé*

*b. Interruption de grossesse*

*c. Test diagnostique invasif*

*d. Traitement à base de cellules souches*

*e. Conseil génétique*

*f. Preuves insuffisantes ou manque de preuves au sujet des options de traitements ci-dessus*

*g. Ne sais pas*

6. Which of the following is the earliest gestational age at which NIPT can be offered to patients?

a. 7 weeks

b. 10 weeks

c. 13 weeks

d. 15 weeks

e. 18 weeks

f. 20 weeks

g. I don’t know

6*. Sous quel délai à partir du début de la grossesse le DPNI peut-il être offert aux patientes ?*

*a. 7 semaines*

*b. 10 semaines*

*c. 13 semaines*

*d. 15 semaines*

*e. 18 semaines*

*f. 20 semaines*

*g. Ne sais pas*

7. All the chromosomal abnormalities diagnosed via amniocentesis can also be readily detected via NIPT.

a. True

b. False

c. I don’t know

*7. Le DPNI permet de détecter aussi facilement qu’une amniocentèse toute anomalie chromosomique.*

*a. Vrai*

*b. Faux*

*c. Ne sais pas*

8. What groups of women should NIPT be offered to? **Select all that apply**

a. All pregnant women

b. Women over the age of 35

c. Women who had another positive prenatal screening test for trisomy

d. Women with a previous pregnancy with fetal aneuploidy

e. Women who can afford to pay for it.

f. Women who would consider termination of pregnancy

g. I don’t know

*8. À quel(s) groupe(s) de femmes le DPNI devrait-il être offert ?* ***Sélectionnez toutes les réponses qui s’appliquent.***

*a. Toute femme enceinte*

*b. Les femmes de 35 ans et plus*

*c. Les femmes ayant testé positif à un autre dépistage prénatal de la trisomie*

*d. Les femmes ayant un antécédent de grossesse avec aneuploïdie fœtale*

*e. Les femmes qui ont les moyens financiers de se le payer*

*f. Les femmes susceptibles de considérer une interruption de grossesse*

*g*. Ne sais pas

9. The most common cause of failed NIPT is low fetal DNA fraction found in maternal serum. Which of the

following factors are associated with a low fetal fraction? **Select all that apply.**

**a.** **Maternal smoking status (*ERRATUM, please see Discussion section for further details*)**

b. Maternal weight

c. Fetal sex

d. Parity

e. Crown-Rump Length

f. Gestational Age

g. I don’t know

*9. L’échec d’un DPNI est principalement dû au faible taux de fraction d’ADN fœtal présent dans le sérum maternel. Le ou lesquels des facteurs suivants peuvent-ils être associés à une faible fraction fœtale ?* ***Sélectionnez toutes les réponses qui s’appliquent.***

*a. Le fait que la mère fume*

*b. Un surpoids maternel*

*c. Le sexe fœtal*

*d. La parité*

*e. La longueur vertex-coccyx*

*f. L’âge de gestation*

*g. Ne sais pas*

10. The current recommendation of the SOGC is that NIPT should be offered…

a) in lieu of invasive testing for women at increased risk of trisomy

b) as a primary screen for all pregnant women

c) As a diagnostic test

d) I don’t k now.

*10. À l’heure actuelle, la SOGC recommande le DPNI :*

*a) Comme alternative aux tests invasifs proposés aux femmes présentant un risque élevé de trisomie*

*b) comme premier test de dépistage pour toute femme enceinte*

*c) Comme test de dépistage diagnostique*

*d) Ne sais pas*

*Your thoughts about NIPT:*

11. For the following questions, please select the number from 1 to 7 on the scale that best describes how you feel at the moment. For example in question 11(a) if you thought your patient having NIPT would be very beneficial, you would circle 1. If you thought it was a slightly beneficial, you would circle 3 and if you thought it was a slightly harmful thing you would circle 5. Please read the scale for each question.

For me, offering NIPT for aneuploidy screening to my patients is:

(a) Beneficial 1 2 3 4 5 6 7 Harmful

(b) Important 1 2 3 4 5 6 7 Unimportant

(c) Bad thing 1 2 3 4 5 6 7 Good thing

(d) Pleasant 1 2 3 4 5 6 7 Unpleasant

*Votre avis sur le DPNI :*

*11. Sur une échelle de 1 à 7, veuillez sélectionner le chiffre qui représente le mieux votre opinion actuelle. Par exemple, pour la question 11(a), si vous pensez qu’il serait très bénéfique à votre patiente d’avoir accès au DPNI, vous devriez encercler le 1. Si vous pensez que le DPNI lui serait moyennement bénéfique, vous devriez encercler le 3 et si vous pensez que le DPNI lui serait néfaste, vous devriez encercler le 5. Veuillez lire l’échelle de chaque question attentivement.*

*Selon moi, offrir le DPNI comme mode de dépistage d’un aneuploïdie est :*

*(a) Bénéfique 1 2 3 4 5 6 7 Néfaste*

*(b) Important 1 2 3 4 5 6 7 Insignifiant*

*(c) Une bonne chose 1 2 3 4 5 6 7 Une mauvaise chose*

*(d) Plaisant 1 2 3 4 5 6 7 Désagréable*

Demographics:

12. Type of practice.

a. Obstetrician/Gynecologist

b. Médecin généraliste

c. Midwife

d. Maternal-fetal-medicine specialist

e. Medical genetics specialist

f. Genetic counselor

g. Nurse

h. Other

i. Prefer not to say

*Données démographiques :*

*12. Type de pratique :*

*a. Obstétricien / Gynécologue*

*b. Médecin généraliste*

*c. Sage-femme*

*d. Spécialiste en médecine fœto-maternelle*

*e. Spécialiste en génétique médicale*

*f. Conseiller en génétique*

*g. Infirmière*

*h. Autre*

*i. Préfère ne pas répondre*

13. Current level of practice.

a. Staff

b. Fellow

c. Resident

d. Student

e. Other

f. Prefer not to say

*13. Présent niveau de pratique :*

*a. Patron*

*b. Fellow*

*c. Interne*

*d. Étudiant*

*e. Autre*

*f. Préfère ne pas répondre*

14. Gender

a. Male

b. Female

c. Other

*14. Sexe:*

*a. Homme*

*b. Femme*

*c. Préfère ne pas répondre*

15. What percentage of your patient population are obstetrical patient?

a. 100%

b. 75-99%

c. 50-74%

d. 25-49%

e. <25%

f. None

g. Prefer not to say

*15. Quel pourcentage de vos patientes consultent pour des questions d’obstétrique?*

*a. 100%*

*b. 75 à 99%*

*c. 50 à 74%*

*d. 25 à 49%*

*e. Moins de 25%*

*f. Aucun(e)*

*g. Préfère ne pas répondre*

16. Years in practice: _____

*16. Nombre d’années de pratique : _____*

17. Province/Territory where you primarily practice?

a. Alberta

b. British Columbia

c. Manitoba

d. New Brunswick

e. Newfoundland and Labrador

f. Nova Scotia

g. Ontario

h. Prince Edward Island

i. Quebec

j. Saskatchewan

k. Northwest Terriroties

l. Nunavut

m. Yukon

*17. Province / Territoire où vous pratiquez principalement ?*

*a. Alberta*

*b. Colombie-Britannique*

*c. Manitoba*

*d. Nouveau-Brunswick*

*e. Terre-Neuve et Labrador*

*f. Nouvelle-Écosse*

*g. Ontario*

*h. Île-du-Prince-Édouard*

*i. Québec*

*j. Saskatchewan*

*k. Territoires du Nord-Ouest*

*l. Nunavut*

*m. Yukon*
